# Early Initiation of Intermittently Scanned Continuous Glucose Monitoring in a Pediatric Population With Type 1 Diabetes: A Real World Study

**DOI:** 10.3389/fendo.2022.907517

**Published:** 2022-06-17

**Authors:** Roberto Franceschi, Vittoria Cauvin, Lorenza Stefani, Federica Berchielli, Massimo Soffiati, Evelina Maines

**Affiliations:** ^1^Pediatric Diabetology Unit, Pediatric Department, S. Chiara General Hospital, Trento, Italy; ^2^Dietology Unit, S. Chiara General Hospital, Trento, Italy

**Keywords:** type 1 diabetes onset, instant scanning continuous glucose monitoring, children and adolescents, outcomes, metabolic control

## Abstract

**Background:**

Use of Continuous Glucose Monitoring (CGM) systems early in the course of diabetes has the potential to help glycemic management and to improve quality of life (QoL). No previous research has examined these outcomes in children-adolescents with type 1 diabetes (T1D) who use intermittently scanned CGM (isCGM) starting within the first month after diagnosis.

**Aim:**

To evaluate the impact of isCGM early after T1D diagnosis, on metabolic control and QoL, comparing a group who started the use of the device within one month from the onset with another one who started at least one year later.

**Subjects and Methods:**

Patients who used isCGM within 1 month from T1D diagnosis were enrolled in group A; those who didn’t have the device during the first year were considered as control group (group B). HbA1c and total daily insulin were evaluated at 3 (T1), 6 (T2) and 12 (T3) months post-baseline (T0, diabetes onset), QoL after 1 year. In group A, isCGM glucose metrics were also recorded.

**Results:**

85 patients were enrolled in group A and 67 patients in group B. In group A isCGM was well accepted during follow up: no patient dropped out; percentage of time with active sensor was in mean > 87%; number of scans/day remained stable. QoL was higher in group A than in group B both in children-adolescents (p<0.0001) and in parents (p 0.003). Group A presented lower HbA1c during the first year after diagnosis (p<0.001), and this data correlated with glucose management indicator (GMI), time in range (TIR) and mean glucose. The honeymoon period lasted more in group A than in B (p 0.028). Furthermore, the mean hypoglycemia duration decreased during follow-up (p 0.001) in group A.

**Conclusions:**

Early use of isCGM, starting within the first month after diagnosis, improves metabolic control and QoL in pediatric patients with T1D.

## Introduction

Children newly diagnosed with type 1 diabetes (T1D) have to adhere to a rigorous and complicated daily medical regimen (1). Improving glycemic control during early months after T1D diagnosis is challenging, because in the honeymoon phase children can experience rapid variations in glucose levels due to unpredictable effects of their own endogenous insulin levels in addition to exogenous insulin administered (2). However, this period is very important to determine future glycemic control and to set habits, beliefs as long as fears (2). Current data suggest that chronically high glucose levels may impair insulin synthesis/secretion, beta cells survival and insulin sensitivity, therefore improving blood glucose (BG) control is the most important step for preserving pancreatic beta cells (3, 4).

Use of Continuous Glucose Monitoring (CGM) systems early in the course of diabetes, has the potential to help glycemic management and to improve quality of life (QoL) (5, 6). However, currently there is a lack of studies about possible outcomes of early initiation of intermittently scanned CGM (isCGM) in pediatric subjects with T1D.

In view of the above, the aim of the study was to measure the impact of early use of isCGM on HbA1c (primary outcome) and QoL (secondary outcome).

## Subjects and Methods

### Ethic Committee

The current study was approved by the Institutional Review Board of “Azienda Provinciale per i Servizi Sanitari della Provincia Autonoma di Trento” (reference number A424). The study was conducted from January 2017 to January 2022. Written informed consent was obtained from each participant and parent/legal guardian, as applicable, prior to enrollment.

### Participants

225 patients with diabetes, aged 1-18 years are followed up at the Pediatric Diabetology Outpatient Clinic of Trento, one of the 68 Italian pediatric diabetes centres belonging to the Italian Society for Pediatric Endocrinology and Diabetes (ISPED) (7).

Inclusion criteria for this study were:

- age between 4-18 years (both inclusive);
- diagnosis of T1D confirmed by the positivity of at least one of the antibodies against islet cells (ICA), insulin (IAA), glutamate dehydroxilase (GADA), islet antigen 2 (IA2A) and zinc-transporter protein 8 (ZnT8A);
- acceptance to wear the isCGM system;
- to stay on the current insulin regimen: multiple daily injection (MDI) or continuous subcutaneous insulin infusion (CSII) for the first year after diagnosis.

Exclusion criteria were:

- age < 4 or > 18 years;
- other forms of diabetes out of T1D;
- use of real-time CGM (rtCGM);
- refusal to wear isCGM;
- change of insulin regimen during the first year after diagnosis (from MDI to CSII or vice-versa);
- diagnosis of developmental delay or severe psychiatric disorder;
- comorbid chronic condition.

### Study Design

IsCGM first-generation (Abbott FreeStyle Libre 1^®^ Glucose Monitoring System) has been available in Italy since 2016, and in our District (Province of Trento), the device was provided for free from January 2017. In the study, patients with new-onset T1D were consecutively enrolled offering to all of them the opportunity to start isCGM first generation soon after diabetes diagnosis (within 1 month). Structured education to the device, to glucose targets and trends was provided by the diabetology staff. Patient who accepted the device were included in group A, while those who didn’t accept the device were excluded from the study.

Patients who presented diabetes onset in the years 2011-2016 that accepted to wear isCGM in 2017, at least 1 year after T1D diagnosis, were considered as control group (group B). For this group data on metabolic control have been retrospectively collected during the first year after diagnosis while QoL at 1 year, as in our centre this parameter is routinely tested.

In group A and B demographic parameters were recorded at diabetes onset (T0), 3 (T1), 6 (T2) and 12 (T3) months post-baseline. Data included: age, anthropometric measurements (height and weight), pubertal status (Tanner stages I-V), total daily insulin dosages, HbA1c. We used HbA1c level as the main indicator of average glycemic control. At diabetes onset we also collected: gender, venous blood pH and serum bicarbonates (HCO3), ethnicity and socioeconomic status (SES).

In group A, from T1 to T3, we recorded isCGM glucose metrics: % of time with active sensor, time in range (TIR), time below range (TBR), coefficient of variation (CV), mean glucose, average number of scans per day, number of hypoglycemia events, mean hypoglycemia duration.

QoL was assessed in children (> 8 years)-adolescents and their parents at 1 year in both groups.

### Methods

Body mass index (BMI) was calculated as weight in kilograms divided by the square of height in metres. BMI-SDS was calculated using the WHO BMI charts (8). Pubertal development was assessed according to Tanner staging (9). Children-adolescents were classified according to three pubertal stages: pre-pubertal (equivalent to the Tanner stage 1), pubertal (Tanner stages 2 to 4), and post-pubertal (Tanner stage 5). Capillary HbA1c level was measured using DCA Vantage^®^ Analyzer (Siemens Healthcare GmbH).

IsCGM data available in the 2-week period preceding every visit were collected, since studies have shown that glucose readings from the last 14 days correlate well with 3 months data and the association between the CGM-measured mean glucose and HbA1c is strong (10–12). QoL was assessed with the Italian version of the PedsQL 3.0 Diabetes Module questionnaire, with the 8-12 years child and parents version, 13-18 years adolescent and parents version (13); range is between 0 to 100, and higher scores indicate higher QoL.

DKA at the diagnosis was defined as venous pH <7.3 or serum bicarbonate <15 mmol/L according to ISPAD guidelines (14). We consider a partial remission or “honeymoon period” according to this definition: HbA1C (%) + [4 * insulin per Kg body weight per day] ≤ 9 (15, 16). The family’s socio-economic status (SES) was evaluated by ascertaining the mother’s educational level. Educational level has been previously identified as an important indicator for SES (17) and was dichotomized into low- (≤14 years of education) and high- (>14 years of education) SES, which differentiates between families with a mother who has completed medium or higher education, college or university training, from other families (18, 19).

### Statistics

In order to determine the optimal number of patients to be consecutively enrolled in the study, when planning the present clinical trial, the calculation of the sample size was performed together with the statisticians. The primary outcome of the study was to identify changes in HbA1c over the first year after diagnosis, between the two groups. We considered a minimum difference of 0,5% and a standard deviation of 0.97 as clinically relevant (5). By accepting a two-tailed 5% α error and a study power of 90% (1-β), a numerosity (n) equal to 86 patients is obtained.

Statistics were analysed using GraphPad Prism version 8.0.2 (GraphPad, San Diego, CA, USA). Every dataset was tested for statistical normality and this information was used to apply the appropriate (parametric or nonparametric) statistical test.

Data are expressed as mean ± SD for variables with normal distribution and with medians (interquartile range) for non-normally distributed variables. Differences between groups of continuous variables were analysed with t-student for paired samples for variables with normal distribution, or with Wilcoxon signed rank sum test for variables with non-normal distribution. The chi-square test with Fisher’s test has been used to evaluate differences in categorical data.

Pearson correlations have been used to analyse statistical relationship between the different variables. P values < 0.05 were considered significant.

## Results

From January 2017 to January 2021, 110 children-adolescents aged 1-18 years were newly diagnosed with T1D at our pediatric clinic. Among them, 16 patients were started on rtCGM (among these, 12 aged less than 4 years were started on CSII + rtCGM) and 4 refused isCGM, therefore they were excluded. 90 subjects accepted to initiate isCGM within 1 month (group A) and 5 more were excluded because they started a pump during the first year after diagnosis.

Among the 108 patients who presented with T1D onset in the years 2011-2016, 78 subjects accepted to wear isCGM in 2017. Eleven were excluded because they had started CSII within the first year after diagnosis, therefore 67 patients were included in group B and their first year after diagnosis was evaluated retrospectively. Population characteristics are reported in **Table 1**; all the subjects were in MDI and nobody started CSII stand-alone or CSII plus isCGM since diagnosis.

**Table 1:** Population characteristic at the T1D onset (T0).

T0	Group A	Group B	p value
**Number**	85	67	–
**Male (%)**	44 (52%)	33 (49%)	n.s (p 0.61)
**Age at onset (years)**	10.95 ± 3.26	10.09 ± 1.68	n.s. (p 0.06)
**DKA at onset (%)**	36 (42%)	25 (37%)	n.s. (p 0.97)
**Age class (years)** 0-4 5-9 10-14 15-19	4 13 19 0	0 16/ 9 0	
**Gender**	17 M/19 F	13 M/12 F	
**Ethnicity** Caucasian Morocco Pakistan-India **SES** High Low	27 7 2 19 17	21 4 0 13 12	
**pH**	7.32 (7.21-7.37)	7.34 (7.18-7.38)	n.s. (p 0.90)
**HbA1c at onset (mmol/mol)**	12.25 ± 2.28	11.8 ± 1.98	n.s (p 0.21)
**Days to isCGM start**	3.82 (2.00-4.00)	809 (422–1177)	**p < 0.0001**
**Ethnicity**	64 Caucasian (75%) 19 Morocco (22%) 2 Indian/Pakistan (2%)	55 Caucasian (82%) 19 Morocco (28%)	n.s. (p 0.82)
**SES**	57 High (67%) 28 Low (33%)	48 High (71%) 19 Low (29%)	n.s. (p 0.55)
**BMI (Kg/m2)**	18.29 ± 2.89	16.89 ± 2.35	n.s. (p 0.07)
**BMI z-score**	-0.51 ± 1.23	-0.29 ± 0.88	n.s. (p 0.23)
**Prepubertal** **Pubertal** **Postpubertal**	46 (54%) 34 (40%) 5 (6%)	41 (61%) 25 (37%) 1 (1%)	n.s. (p 0.74)

Group A included patients that accepted to initiate isCGM within 1 month from T1D diagnosis. Group B included patients that started the device at least 1 year after diagnosis.

Age at onset, HbA1c at onset, weight, BMI, and BMI z-score are expressed as mean ± SD.

pH and days to isCGM start are expressed as median ± interquartile range.

IsCGM, Intermittently Scanned Continuous Glucose Monitoring; DKA, diabetic ketoacidosis. SES, socio-economic status; n.s., not significant. In bold: statistically significant p values.

### Data at T1D Onset

Age and diabetes onset severity in terms of pH, % of DKA and HbA1c were similar in the 2 groups, as well as gender distribution, ethnicity, SES, BMI z-score and pubertal status (**Table 1**). Patients in group A started isCGM at a mean of 3.82 days after diabetes diagnosis, while in group B after a mean of 832 days from T1D diagnosis.

### Follow up Data

No events of severe hypoglycemia or recurrence of DKA occurred during the study, and no patients dropped out from isCGM. No skin reactions occurred due to the use of isCGM systems during the study period. Patients with isCGM (group A) showed lower HbA1c levels than group B at any time (p<0.01) (**Table 2**). HbA1c increased both in group A and B from 3 to 6 and 12 months (group A: p < 0.0001, group B p<0.05) as well as the number of patients in the honeymoon period decreased during the first year after diagnosis at any time, with longer honeymoon duration in patients with isCGM (group A) than in group B (36,5% in honeymoon period at 12 months compared to 14.92% in group B, p 0.0028). Considering only patients who entered remission within the first 3 months, the number of patients in the honeymoon period progressively decreased during the first year after diagnosis in both groups, with group A maintaining longer honeymoon duration than group B (61% in honeymoon period at 12 months compared to 43% in group B, p 0.028).

**Table 2:** follow up data in Group A (n = 85) and B (n = 67).

Parameters	Group	T1 (3mo)	T2 (6mo)	T3 (12mo)	p value
**HbA1c (%)**	A B	6.45 ± 0.47 6.86 ± 0.71 **p < 0.0001**	6.58 ± 0.64 7.00 ± 0.72 **p 0.0002**	6.88 ± 0.72 7.19 ± 0.76 **p < 0.001**	A: **p < 0.0001** B: **p < 0.05**
**Total daily insulin dose (U/Kg)**	A B	0.43 ± 0.25 0.45 ± 0.22 n.s. p 0.58	0.44 ± 0.24 0.49 ± 0.23 n.s. p 0.25	0.47 ± 0.30 0.52 ± 0.22 n.s. 0.29	A: n.s. p 0.55 B: n.s. p 0.21
**% of basal insulin**	A B	61.07 ± 20.84 58.1 ± 15.42 n.s. p 0.33	59.93 ± 21.55 57.10 ± 15.31 n.s. p 0.36	60.96 ± 19.05 53.63 ± 14.95 **p 0.01**	A: n.s. p 0.92 B: n.s. p 0.21
**Number of patients in “honeymoon period”** (all patients)	A B	53/85 (62%) 29/67 (43%) **p 0.019**	38/85 (44.7%) 16/67 (23.9%) **p 0.0075**	31/85 (36.5%) 10/67 (14.92%) **p 0.0028**	A: **p 0.0024** B: **p 0.0007**
**Number of patients in “honeymoon period”** (patients entered remission within the first 3 months)	A B	65/85 (76%) 43/67 (64%) n.s. p 0.098	59/85 (69%) 36/67 (54%) **p 0.0047**	52/85 (61%) 29/67 (43%) **p 0.028**	A: **p 0.098** B: **p 0.05**
**BMI z-score**	A B	-0.27 ± 1.04 -0.07 ± 0.80 n.s. p 0.20	-0.29 ± 1.01 -0.20 ± 0.81 n.s. p 0.57	-0.21 ± 1.04 -0.16 ± 0.84 n.s. p 0.78	A: n.s. p 0.86 B: n.s. 0.61
**Patients PedsQL score**	A B	n.a.	n.a.	83.14 ± 7.87 74.58 ± 9.29 **p < 0.0001**	
**Parents PedsQL score**	A B	n.a.	n.a.	79.78 ± 10.21 73.68 ± 14.68 **p 0.003**	

Data are presented as mean ± SD. N.a., not assessed.

n.s., not significant.

In bold: statistically significant p values.

No significant differences in total daily insulin dose and in BMI z-score were showed during follow up at any time (T1, T2, T3) in group A and in group B and between groups. QoL at 1 year was statistically significant higher in children in group A than in group B (83.14 ± 7.87 vs 74.58 ± 9.29, p < 0.0001), as well as in parents (79.78 ± 10.21 vs 73.68 ± 14.68, p 0.003).

### IsCGM Metrics in Group A

During follow up the percentage of time with active sensor slightly decreased (p 0.03), and CV increased at any time (p 0.0036, **Table 3**). Number of hypoglycemia events per week remained stable, while the mean hypoglycemia duration decreased from 3 months to 6 and 12 months (p 0.01).

**Table 3:** four week time glucose metrics in Group A (n = 85).

Parameters	T1 (3mo)	T2 (6mo)	T3 (12mo)	p value
**% of time with active sensor**	91.9 ± 9.12	90.65 ± 9.53	87.55 ± 13.10	**p 0.03**
**% of time in range (70-180 mg/dL)**	72.26 ± 11.59	70.82 ± 12.04	68.52 ± 12.51	n.s. p 0.13
**% di time below range (< 70 mg/dL)**	4.93 ± 3.51	4.74 ± 3.65	5.18 ± 4.18	n.s. 0.75
**Mean glucose (mg/dL)**	139.49 ± 13.89	141.21 ± 18.05	144.13 ± 18.16	n.s. 0.19
**Coefficient of variation (CV) (%)**	32.94 ± 6.65	35.30 ± 8.22	36.74 ± 7.16	**p 0.0036**
**Glucose management indicator (GMI) (%)**	6.65 ± 0.43	6.68 ± 0.62	6.73 ± 0.58	n.s. 0.64
**Number of hypoglycemic events/week**	7.36 ± 3.80	6.74 ± 4.12	7.73 ± 4.35	n.s. 0.28
**Mean hypoglycemia duration (min)**	52.85 ± 26.79	39.42 ± 24.62	41.49 ± 23.85	**p 0.001**
**Average number of scans per day**	10.53 ± 6.10	9.86 ± 5.52	10.19 ± 5.35	n.s. 0.74

Data are presented as mean ± SD. n.s., not significant.

In bold: statistically significant p values.

No significant differences in TIR, TBR, mean glucose and GMI were registered. Average number of scans per day did not change along the follow up period.

### Correlations

HbA1c strongly correlated with TIR, mean glucose and GMI at any time (**Table 4**).

**Table 4:** correlations among HbA1c and study variables at T1, T2 and T3 in group A.

Parameters	HbA1c atT1 (3mo)	HbA1c atT2 (6mo)	HbA1c atT3 (12mo)
**% of time with active sensor**	n.s. p 0.60	n.s. p 0.07	n.s., p 0.064
**% of time in range (70-180 mg/dL)**	**p 0.003,** r –0.32	**p < 0.0001**, r -0.61	**p < 0.0001**, r -0.45
**% di time below range (< 70 mg/dL)**	n.s. p 0.51	**p < 0.0001**, r 0.43	n.s., p 0.28
**Coefficient of variation (CV) (%)**	n.s. p 0.11	n.s. p 0.22	n.s. p 0.078
**Mean glucose (mg/dL)**	**p 0.001,** r 0.36	**p < 0.0001**, r 0.43	**p < 0.0001**, r 0.67
**Glucose management indicator (GMI) (%)**	**p < 0.0001**, r 0.37	**p < 0.0001**, r 0.77	**p < 0.0001**, r 0.72
**Number of hypoglycemic events/week**	n.s. p 0.98	**p 0.002,** r 0.33	n.s. p 0.62
**Mean hypoglycemia duration (min)**	n.s. p 0.87	**p 0.013**, r 0.27	n.s. p 0.92
**Average number of scans per day**	**p 0.004**, r – 0.31	n.s. p 0.96	n.s. 0.41

ns, not significant.

In bold: statistically significant p values.

TIR strongly correlated with HbA1c, GMI, time of sensor usage, mean glucose and CV (**Supplementary Material S1**).

## Discussion

We studied the impact of isCGM, started within 1 month from T1D onset, on HbA1c and QoL, as no reports on isCGM in relation to diabetes onset are available in literature (20). According to us three are the main findings:

1. Early initiation of isCGM within 1 month after T1D diagnosis, is feasible and well accepted, as demonstrated by the absence of dropout among our cohort and by the improved QoL in children as well as in parents. It is safe as no events of severe hypoglycemia or recurrence of DKA occurred during the study. Moreover, isCGM did not increase total daily insulin dosage or BMI z-score. Our data are concordant with previous studies about early initiation of rtCGM (from 0 to 12 months), that demonstrated that it is feasible and well accepted by youth and their families (6). In our study compliance with wearing the instrument was high (mean > 87%) and the percentage of time with active sensor just slightly decreased during the first year of follow up, in agreement with previous studies on rtCGM at T1D diagnosis (21). Such findings are important as we know from literature that frequent sensor usage (at least 70%) is associated with greatest improvement in glycemic control in patients with CGM (22). Our patients were compliant to scan the device: an average number of 10 scans per day were maintained during follow up, as we suggested at the start of the device by structured education (before the three main meals, before snacks, before going to bed, in case of symptoms of hyper or hypoglycemia, and for physical activity management).
2. The main outcome of our study was HbA1c. Patients who started isCGM within 1 month after T1D diagnosis (group A) had lower HbA1c at 3, 6 and 12 month follow-up compared to those who did not start early isCGM (group B). The improvement in HbA1c levels within the first 12 months of diabetes is similar to that reported for rtCGM (5). Furthermore, we confirm a correlation between the 2 weeks sensor metrics (TIR, mean glucose, CV, GMI) with 3 months data and HbA1c, as other studies report (10–12).

HbA1c is strongly correlated with GMI, TIR and mean glucose and this data, linked to the use of isCGM during the first year after diagnosis, have never been reported in literature before. We interpreted this data, considering that this device allows subjects to have more information about glucose values, trends and to look at patterns. According to this, they can enhance insulin dosage, food intake and physical activity, improving TIR and HbA1c too. At the same time, having more glucose data gradually increases confidence in the device as they could improve hypoglycemia correction, leading to reduction in mean hypoglycemia duration, as we found from 3 to 6 and 12 months. Vice-versa, as we expected, isCGM cannot prevent a number of hypoglycemia events, unlike rtCGM systems (21).

3) We found that even in group A, HbA1c levels increased between 3 months and the 6-12 months follow-up assessments as well as the coefficient of variation for glucose, a measure of glycemic variability. These data are generally consistent with the findings of other authors regarding rtCGM (5, 23, 24), probably due to the ending of partial remission period (honeymoon) in some patients. Interestingly, the percentage of patients that remained in the honeymoon period was higher in the isCGM group compared to group B, probably related to better HbA1c and values of glucose on target. Recent evidence points to the usefulness of new technologies like rtCGM and insulin pumps to improve metabolic control, as this may preserve C-peptide and other outcomes, both with and without additional immunomodulatory therapy at the onset of T1DM (25).

Strengths of the present study are: i) this is a real world study, all the patients with diabetes onset had the opportunity to wear isCGM because private insurance was not required. Low socio-economic groups had the same opportunity to have access to CGM systems. Therefore, the present study can be generalised to other cohorts of children and adolescents with recent-onset T1D in a universal health care system; ii) all the patients enrolled resulted in MDI, then variables due to other technologies are excluded. The main limitation to the present study is that it was conducted at a single site and other studies are needed to confirm whether introduction the of isCGM devices, early in the course of diabetes along with education around glucose targets, has the potential to improve glycemic outcomes and QoL.

## Data Availability Statement

The raw data supporting the conclusions of this article will be made available by the authors, without undue reservation.

## Ethics Statement

The studies involving human participants were reviewed and approved by Institutional Review Board of “Azienda Provinciale per i Servizi Sanitari della Provincia Autonoma di Trento” (reference number A424). Written informed consent to participate in this study was provided by the participants’ legal guardian/next of kin.

## Author Contributions

RF designed the study. LS, FB, and EM researched and analysed data. RF, VC, MS, and EM wrote the manuscript. RF is the guarantor of this work and, as such, has full access to all the data in the study and takes responsibility for the integrity and the accuracy of the data analysis. All authors contributed to the article and approved the submitted version.

## Conflict of Interest

The authors declare that the research was conducted in the absence of any commercial or financial relationships that could be construed as a potential conflict of interest.

## Publisher’s Note

All claims expressed in this article are solely those of the authors and do not necessarily represent those of their affiliated organizations, or those of the publisher, the editors and the reviewers. Any product that may be evaluated in this article, or claim that may be made by its manufacturer, is not guaranteed or endorsed by the publisher.

## Abbreviations

CGM, Continuous glucose monitoring; isCGM, Intermittently scanned continuous glucose monitoring; rtCGM, Real time continuous glucose monitoring; T1D, Type 1 diabetes; DKA, Diabetic ketoacidosis; HbA1c, Glycated hemoglobin (glycohemoglobin, hemoglobin A1c); TIR, Percentage of time in range 70-180 mg/dL (3.9 – 10 mmol/L); TBR, Percentage of the time below the range <70 mg/dL (3.9 mmol/L); CV, Coefficient of variation; GMI, Glucose management indicator; BG, blood glucose; QoL, quality of life; SES, socio-economic status.
